# Validity Concerns About the Heartbeat Counting Task Extend to Alcohol Use disorder: Evidence From Subclinical and Clinical Samples

**DOI:** 10.1111/adb.70032

**Published:** 2025-05-01

**Authors:** Pauline Billaux, Olivier Desmedt, Olivier Corneille, Olivier Luminet, Mateo Leganes‐Fonteneau, Joël Billieux, Pierre Maurage

**Affiliations:** ^1^ Louvain Experimental Psychopathology Research Group (LEP) Psychological Science Research Institute, UCLouvain Louvain‐la‐Neuve Belgium; ^2^ Fund for Scientific Research (FRS‐FNRS) Brussels Belgium; ^3^ Institute of Psychology University of Lausanne Lausanne Switzerland; ^4^ The Swiss National Science Foundation (SNSF) Berne Switzerland; ^5^ Psychological Science Research Institute, UCLouvain Louvain‐la‐Neuve Belgium; ^6^ Center for Excessive Gambling, Addiction Medicine Lausanne University Hospital (CHUV) Lausanne Switzerland

## Abstract

Theoretical models propose that interoception plays a role in addictive disorder. However, this assumption has been mostly tested using the heartbeat counting task (HCT), which is known to be contaminated by estimation strategies. An adapted version of the HCT (in which respondents report only *felt* heartbeats) has been developed to reduce estimation biases. Here, we examined the validity of the classical and adapted HCT versions in samples presenting alcohol use disorders. We recruited a clinical sample of 48 patients with severe alcohol use disorder (SAUD), matched with 41 healthy controls (HC), and a subclinical sample of 32 binge drinkers (BD), matched with 30 HC. Participants performed the classical HCT, adapted HCT, and a time estimation task. We additionally assessed mental health variables theoretically related to interoception (alexithymia, anxiety, childhood trauma, depression and emotion regulation). In all groups, HCT scores were smaller in adapted than classical HCT. Patients with SAUD, but not BD, showed lower HCT scores than matched controls, independently of the task. We found no correlation between HCT scores and psychological constructs. Heartbeats reported during classical HCT correlated with seconds reported during time estimation task for SAUD and matched HC, suggesting the use of time estimation strategies to perform the task. The largely reduced HCT performance in the adapted version, the association between HCT performance and time estimation performance and the lack of theoretically expected associations between HCT scores and psychological variables extend doubts on the validity of these tasks for measuring interoceptive accuracy in problematic alcohol consumption.

## Graphical abstract

Interoception might play a role in addiction, but this assumption has only been tested using the widely criticised heartbeat counting task (HCT). We explored HCT validity in severe alcohol use disorder and binge drinking, through a classical/adapted HCT. Our results extend doubts on HCT validity to alcohol‐related disorders as we showed (1) largely reduced HCT performance in the adapted version; (2) links between HCT and time estimation; (3) a lack of theoretically expected associations between HCT performance and psychological variables.

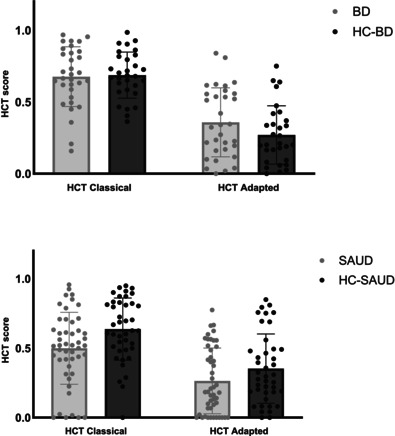

## 1 Introduction

Interoception, defined as the representation of the organism internal state, encompasses a set of bottom‐up and top‐down processes (i.e., perception, integration and interpretation of internal bodily states). These processes, at both unconscious and conscious levels, allow internal states to be communicated to, processed, and anticipated by the brain, generating an integrated representation of body states [1]. Theoretical models have suggested that interoception plays a role in substance use disorders (SUD) onset and maintenance [2], at unconscious level. Interoception role has been notably emphasized at the cerebral level in the triadic model of addiction [3], postulating that insular (neural interoceptive hub) processing of bodily states increases the imbalance between hypoactivated prefrontal cortex and hyperactivated amygdala‐striatal system, favouring immediate drug consumption despite negative consequences [3]. Neuroimaging studies have concurred on insula role in SUD, reporting insular preferential pattern of activation when processing drug‐related cues [4]. For example, an anterior insula hyperactivation has been found in individuals at risk for cannabis use disorder, when exposed to cannabis odour [5].

At the behavioural and more conscious level, models also posit a role of interoception in SUD (see [6, 7] for an illustration in AUD). In particular, conditioning models theorize incentives for substance use as means to reduce negative affect or withdrawal symptoms (negative reinforcers) and to enhance positive affect or pleasure during consumption (positive reinforcers) [2]. According to such models, associations between drug reinforcers and their effects on bodily states emerge from learning mechanisms [2, 8]. This learning is thought to be supported by interoceptive processes, as internal signals are consciously associated with psychoactive substances effects [2, 8].

Interoception thus holds promise as a critical dimension in the aetiology of SUD at both unconscious and conscious levels, with behavioural measures of interoception needing integration into basic research to clarify its relevance in addiction. Most of these measures are meant to index one's ability to detect bodily states (i.e., interoceptive accuracy) and could help elucidate individual differences supporting adaptive and maladaptive mechanisms in addiction. Previous research shows that alcohol impacts a range of physiological systems, including the cardiovascular system, both acutely [9] and chronically [10], which could feed into addiction development and persistence. Sensitivity to these physiological changes, as indexed by behavioural interoceptive measures, could thus reveal etiological mechanisms in addiction. For instance, alcohol acute effects on cardiac interoception correlate with subjective intoxication [11] and long‐term expectancies [12] in social drinking. These findings, along with broader research on sensitivity to alcohol effects as an addiction risk marker [13], suggest that interoception may influence the onset and maintenance of problematic alcohol consumption pattern. They also position cardiac interoception as one of the relevant constructs for measuring sensitivity to bodily processes across the continuum of alcohol use, from subclinical to clinical populations.

Interoception may thus represent a relevant factor in SUD aetiology, but assessing interoceptive abilities is characterized by several challenges [14]. The first challenge resides in the focus on cardiac measures of interoception—and the addiction field makes no exception. While ethanol has acute and long‐term effects on the heart, the sole focus on this sensory‐axis can be questioned as ethanol impacts a large variety of organs [10]. The assessment of interoceptive accuracy in problematic alcohol use should thus evolve towards other sensory‐axes, as they rely on different neural substrates [15] and their accuracy measures do not appear to correlate [16]. The second challenge is related to the validity of the most widely used measure of interoceptive accuracy in severe alcohol use disorder (SAUD), the heartbeat counting task (HCT) [7]. This task focuses on cardiac signals, which are easily measurable and less susceptible to participant's control [14, 17]. Higher ability to detect these bodily signals would reflect higher cardiac interoceptive accuracy. In the HCT, participants count their heartbeats at rest, without taking their pulse, during defined time intervals, while actual heartbeats are recorded. This allows computing an index of cardiac interoceptive accuracy, by comparing actual against reported heartbeats [18]. However, this task is facing major backlash due to its evidenced lack of construct validity [14, 19]. First, HCT performance is contaminated by guessing strategies (e.g., time estimation [20]). Second, recent meta‐analyses failed to report associations between HCT performance and theoretically related mental health variables [21, 22]. Finally, studies using the HCT are not necessarily comparable due to lack of standardization, which compromises reproducibility [19]. For instance, in populations diagnosed with SAUD, empirical studies showed major discrepancies in instructions used [23, 24], as well as in duration and number of time intervals [24, 25].

Despite widespread criticism of the HCT, it remains to date the main measure of interoceptive accuracy used in the few studies conducted in SAUD. Findings from these studies have led authors to propose therapeutical venues for patients based on a performance at a task whose validity is widely criticized [26]. Reliance on the HCT may have lasting implications for how future addiction researchers conceptualize and measure interoception.

Our main goal is to assess what can be reliably inferred from the HCT in the context of problematic alcohol consumption, by testing the construct validity of the HCT in SAUD and in a subclinical group of binge drinkers (BD). The continuum hypothesis proposes that BD and SAUD share qualitatively similar impairments at cognitive and cerebral levels (such impairments being quantitatively greater in SAUD [27]), and testing SAUD and BD simultaneously thus allows observing changes in interoception across the continuum of alcohol use. This was achieved through three objectives. First, we tested the construct validity of the classical HCT in patients with SAUD and subclinical BD. To do so, we followed Desmedt et al.'s [28] protocol, which consists in asking participant to perform the classical HCT (i.e., participants are merely asked to count their heartbeats) and the adapted HCT (i.e., participants are instructed to report solely *felt* heartbeats, without guessing). Using such protocol in a normative sample, the authors found a 50% reduction in HCT performance in the adapted task, therefore supporting the substantial contribution of non‐interoceptive processes to task performance in the classical version of the HCT. In our study, we expected that if the HCT were valid in BD and SAUD populations, the instructions would have no influence on HCT scores as participants would only focus on felt heartbeats in both versions of the task. Conversely, if the HCT were to prove invalid, we would expect that scores at the adapted HCT would be lower than those obtained at the classical HCT across all participants [28], because participants would less rely on non‐interoceptive strategies in the adapted task, such strategies artificially increasing performance at the classical HCT. Regarding the group differences in HCT scores between SAUD and BD groups, we expected lower scores among BD and SAUD populations (vs. HCs) on the classical version of the task (as previously observed), but we had no expectation regarding the adapted HCT.

Second, we evaluated the relationship between HCT performance, psychological constructs and psychiatric variables (assessed with self‐reported measures) previously examined in relation to cardiac interoceptive accuracy in SAUD: alexithymia [26], anxiety symptoms [23], childhood trauma [29], depressive symptoms [23], dissociative symptoms [30], emotion regulation [31], self‐reported interoceptive sensitivity to mostly uncomfortable physiological sensations [23] and sleep problems [23]. We hypothesized that if the classical HCT holds concurrent validity in addiction, we would expect that HCT scores correlate with scores on self‐reported questionnaires assessing psychopathological constructs and psychiatric variables typically associated with interoception impairments [23, 26, 29, 30, 31]. Conversely, if the HCT is invalid, we would expect no association between interoceptive accuracy and the questionnaires scores.

Third, we explored the contribution of time estimation strategies in HCT performance by asking participants to complete a procedurally matched time estimation task during which they had to count the seconds of each interval [20]. We hypothesized that, would the HCT be valid, we should observe no association between heartbeats reported during this task and seconds reported during the time estimation task. Conversely, a positive correlation would suggest the use of non‐interoceptive strategies when performing the HCT [20] and, hence, would reveal the low validity of this task.

## 2 Materials and Method

### 2.1 Participants

We included two samples of individuals with problematic alcohol consumption, namely, a clinical sample of recently detoxified patients with SAUD and a subclinical sample of BD, both matched with a group of healthy controls (HC‐BD and HC‐SAUD). The study protocol followed the Declaration of Helsinki's ethical standards and was approved by the local Ethics Committee of the Psychiatric Hospital of Beau Vallon and the Ethics Committee of the Psychological Sciences Research Institute (IPSY).

#### 2.1.1 SAUD and HC‐SAUD

The sample included 48 SAUD patients (22 females) and 41 HC‐SAUD (24 females). Patients were diagnosed with SAUD, according to the DSM‐5 criteria (≥ 6 diagnosis criteria [32]). Participants were all recruited from an inpatient treatment unit (Psychiatric Hospital of Beau Vallon, Belgium) and had abstained from alcohol for six to 52 days. Alcohol consumption prior to detoxification was measured using the Timeline Followback Assessment Method [33]. HC‐SAUD were recruited online (i.e., social networks and emails) and through flyers displayed in different locations around the University (Louvain‐la‐Neuve, Belgium). HC‐SAUD had first to fill in an online questionnaire (through Qualtrics Software; Qualtrics LLC, Provo, UT) assessing sociodemographic and alcohol‐related variables to ensure that they were eligible to participate in the study. Inclusion criteria for HC‐SAUD were (1) having no personal or familial past/current SAUD diagnosis; (2) reporting low alcohol consumption (i.e., score < 8 at the Alcohol Use Disorders Identification Test, AUDIT [34]); and (3) drinking less than 11 standard alcohol units per week and four units per day. Exclusion criteria shared by both groups were polysubstance use (excluding nicotine) and major psychiatric and neurological diagnosis. HC‐SAUD had to refrain from drinking alcohol for at least 3 days prior to testing, to control for ethanol acute effects.

#### 2.1.2 BD and HC‐BD

The sample comprised 32 BD (15 females) and 30 HC‐BD (15 females) participants. All participants were recruited online (i.e., social networks and emails) and asked to complete a Qualtrics survey (Qualtrics Software; Qualtrics LLC, Provo, UT) to ensure eligibility. BD participants were selected if they matched Maurage et al.'s [35] integrated conceptualization of BD, based on six criteria including both drinking threshold and continuum criteria over a period of 12 months: (1) report of physiological BD episodes (i.e., blood alcohol consumption threshold of 0.08%); (2) report of psychological BD episodes (i.e., self‐reported feelings of drunkenness); (3) physiological and/or psychological BD episodes represent at least 30% of the drinking episodes; (4) physiological and/or psychological BD episodes occur at least twice per month; (5) physiological BD episodes are reached within 2–3 h; (6) at least 3 days of abstinence per week. Moreover, BD had to present a minimal score of 24 at Townshend and Duka's [36] binge‐drinking score. Inclusion and exclusion criteria for HC‐BD also derived from Maurage et al. [34]. They were eligible if their AUDIT [33] scores were lower than 8 and they did not report BD episode in the past 12 months, nor lifetime regular BD episodes. BD and HC‐BD shared the same exclusion criteria than SAUD and HC‐SAUD, as well as the absence of personal or familial past/current SAUD diagnosis. Both HC‐BD and BD groups had to abstain from alcohol 3 days prior to testing.

### 2.2 Procedure and Measures

Prior to testing, participants were informed about the study procedure and provided written consent for their participation. Participants were not made aware of hypotheses until the experimental session end. Participants put a Polar Watch RS800CX heart monitor's belt around their chest. Such apparatus provided an objective measure of the number of heartbeats within each time interval of the tasks. Participants performed, in a fixed order, the (i) classical HCT; (ii) adapted HCT; (iii) time estimation task (i.e., measure of non‐interoceptive strategy). This order was chosen to control for potential biasing effects of instructions on task performance [28]. Each task was composed of three‐time intervals of 25, 35 and 45 s (counterbalanced order). The beginning and end of each time interval was notified by an auditory cue produced by the Polar Watch. Each time interval was separated by a break of 20 s. Written instructions were provided for the first two tasks. They were the exact replica of the ones used in Desmedt et al. [28]. Instructions for the classical task required participants to count their heartbeats without trying to feel their neck or wrists pulses. In the adapted task, equivalent instructions were given. In addition, participants had to rely only on interoceptive signals and refrain from using non‐interoceptive strategies (i.e., time estimation strategies or heartbeats guessing). Contribution of time estimation strategies (i.e., measure of non‐interoceptive strategy) was assessed using a time estimation task. For the time estimation task, participants were verbally asked to count silently the time in seconds that composed each interval. They were then instructed to report orally the counted seconds, following the auditory cue that notified the end of the time interval.

In addition to these measures, a set of complementary self‐reported mental health variables that had been previously related to cardiac interoceptive accuracy impairments in SAUD [23, 26, 29, 30, 31] were assessed through validated questionnaires: alexithymia (20‐item Toronto Alexithymia Scale, TAS‐20 [37]), anxiety symptoms (State and Trait Anxiety Inventory, form A and B, STAI‐A and B [38]), childhood trauma (Childhood Trauma Questionnaire, CTQ [39]), depression symptoms (Beck Depression Inventory, BDI‐13 [40]), dissociative symptoms (Dissociative Experiences Scale, DES [41]), emotion regulation (Difficulties in Emotion Regulation Scale, DERS [42]), self‐reported awareness and sensitivity to mostly uncomfortable physiological sensations (Private Body Consciousness subscale, PBCS [43]) and sleep problems (Athens Insomnia Scale, AIS [44]). Then, participants were debriefed, and both HC groups and BD participants received a monetary compensation of 10 euros for their participation.

### 2.3 Statistical Analyses

We performed statistical analyses separately for each of our two samples (i.e., SAUD and HC‐SAUD and BD and HC‐BD). We conducted all statistical analyses on Jamovi 2.2 [45, 46], using a significance level of alpha 0.05 (bilateral).

We conducted between‐group comparisons (i.e., independent‐samples *t*‐tests and chi‐square tests of independence) on demographic measures, alcohol‐related variables and questionnaires assessing clinical and psychological variables. When samples quantitative data had equal variances (assessed through Levene's homogeneity of variance test) and were normally distributed (assessed through Shapiro–Wilk normality test), we conducted independent‐samples *t*‐tests. When we found unequal variances but normal distribution of the data, we conducted Welch's tests instead. When we found the data to be abnormally distributed, we conducted Mann–Whitney *U* tests.

To compute an index of interoceptive accuracy for each participant in each condition (i.e., classical and adapted HCT), we used the following formula: 1/3 Σ (1–(|actual heartbeats–reported heartbeats|)/actual heartbeats) [28]. Higher scores corresponded to higher interoceptive accuracy. We used the resulting values to conduct two (i.e., HC‐SAUD/SAUD and HC‐BD/BD) linear mixed model analyses, where the dependent variable was the computed score, the factors were task and group, and participants were the random factor. Significant main effects were followed by Bonferroni post hoc tests.

To investigate time estimation strategy, we computed two variables: (1) counted seconds per minute; 1/3 Σ ((reported seconds/time interval)*60) and; (2) reported heartbeats per minute; 1/3 Σ ([reported heartbeats/time interval]*60) [20]. We then conducted Pearson correlations using these variables, in a within‐group manner.

Finally, we conducted exploratory analyses regarding the association (Spearman's correlation) between HCT scores and scores at the questionnaires, in a within‐group manner. Due to the high number of comparisons, we corrected for multiple comparisons following the Benjamini–Hochberg procedure, using a false discovery rate of 5%.

## 3 Results

### 3.1 Sample Characteristics

#### 3.1.1 SAUD and HC‐SAUD (Table 1)

**TABLE 1:** Sociodemographic, alcohol‐related and clinical variables of patients with severe alcohol use disorder (SAUD) and their paired healthy controls (HC‐SAUD).

	HC‐SAUD (*n* = 41)	SAUD (*n* = 48)	Group comparison test values
**Sociodemographic variables**			
Age	43.98 (10.97)	47.10 (9.47)	*t*(87) = 1.44, *p* = 0.15
Education level	16.12 (2.17)	12.90 (2.57)	*U* = 334.50, *p* < 0.001
Gender, male (%)	41.00	54.00	χ^2^ (1, 89) = 1.43, *p* = 0.23
**Alcohol‐related variables**			
Abstinence duration (in days)	N/A	19.09 (9.64)	/
Age at first alcohol consumption (years old)	15.32 (2.40)	15.32 (4.63)	*U* = 912.50, *p* = 0.69
Consumption per drinking occasion (in standard units)	1.88 (1.30)	19.92 (9.15)	*U* = 0.00, *p* < 0.001
Severity of alcohol use disorder (Number of DSM‐5 criteria)	N/A	8.06 (2.12)	/
AUDIT	3.02 (1.77)	N/M	/
**Clinical variables**			
AIS	5.00 (3.55)	10.17 (5.84)	*t*(78.95) = 5.02, *p* < 0.001
BDI	3.73 (3.79)	12.58 (7.35)	*U* = 238.00, *p* < 0.001
CTQ—Denial	0.56 (1.05)	0.48 (0.81)	*U* = 798.00, *p* = 0.73
CTQ—Emotional abuse	8.03 (3.33)	9.53 (6.17)	*U* = 785.50, *p* = 0.82
CTQ—Emotional neglect	10.07 (3.94)	13.37 (6.87)	*U* = 636.00, *p* = 0.07
CTQ—Physical abuse	6.56 (3.62)	7.11 (4.20)	*U* = 785.00, *p* = 0.77
CTQ—Physical neglect	6.33 (2.63)	8.72 (4.36)	*U* = 530.50, *p* = 0.003
CTQ—Sexual abuse	5.46 (1.10)	7.67 (5.05)	*U* = 681.50, *p* = 0.11
DES	11.48 (7.71)	23.19 (15.06)	*t*(70.09) = 4.57, *p* < 0.001
DERS‐F—Total	69.83 (18.52)	100.60 (26.14)	*t*(82.43) = 6.40, *p* < 0.001
DERS‐F—Awareness	16.05 (8.77)	18.62 (5.17)	*U* = 624.00, *p* = 0.007
DERS‐F—Clarity	9.60 (3.04)	13.64 (4.33)	*U* = 411.00, *p* < 0.001
DERS‐F—Goals	12.22 (4.60)	15.64 (4.78)	*t*(85) = 3.38, *p* = 0.001
DERS‐F—Impulse	9.35 (4.21)	14.13 (5.78)	*U* = 432.50, *p* < 0.001
DERS‐F—Nonacceptance	8.75 (5.46)	16.87 (6.46)	*t*(85) = 6.36, *p* < 0.001
DERS‐F—Strategies	13.85 (6.09)	21.70 (6.74)	*t*(85) = 5.66, *p* < 0.001
PBCS	11.99 (3.43)	12.28 (3.19)	*U* = 711.00, *p* = 0.49
STAI‐A	30.27 (7.62)	38.81 (12.85)	*U* = 576.00, *p* < 0.001
STAI‐B	38.17 (10.06)	53.95 (11.08)	*t*(85) = 6.92, *p* < 0.001
TAS	41.00 (10.42)	55.57 (12.87)	*t*(81) = 5.54, *p* < 0.001
TAS—Difficulty describing feelings	12.11 (4.20)	15.91 (4.24)	*t*(81) = 4.06, *p* < 0.001
TAS—Difficulty identifying feelings	13.19 (4.14)	21.12 (6.78)	*t*(77.53) = 6.57, *p* < 0.001
TAS—Externally oriented thinking	15.69 (4.34)	18.53 (4.94)	*t*(81) = 2.73, *p* = 0.008

Abbreviations: AIS, Athens Insomnia Scale; AUDIT, Alcohol Use Disorders Identification Test; BDI, Beck Depression Inventory; CTQ, Childhood Trauma Questionnaire; DERS‐F, Difficulties in Emotion Regulation Scale‐French; DES, Dissociative Experiences Scale; DSM, Diagnostic and Statistical Manual of Mental Disorders; N/A, Not Appropriate; N/M, not measured; PBCS, Private Body Consciousness subscale; STAI, State (‐A) and Trait (‐B) Anxiety Inventory; TAS, Toronto Alexithymia Scale.

Concerning alcohol‐related measures, SAUD patients did not differ from HC‐SAUD regarding the age at which they started drinking alcohol, but currently consumed more alcohol units per drinking occasion than HC‐SAUD.

Regarding psychological and clinical variables, patients with SAUD reported more sleep problems and depressive symptoms, experienced more physical neglect during their childhood, experienced more dissociative symptoms and difficulties at regulating emotions, had higher state and trait anxiety and higher alexithymia levels than HC‐SAUD. We found HC‐SAUD and SAUD to have experienced nonsignificantly different levels of childhood trauma denial, emotional abuse, emotional neglect, physical abuse, sexual abuse and to have similar levels of awareness and sensitivity to mostly uncomfortable physiological sensations, as measured via the PBCS.

#### 3.1.2 BD and HC‐BD (Table 2)

**TABLE 2:** Sociodemographic, alcohol‐related and clinical variables of binge drinkers (BD) and their paired healthy controls (HC‐BD).

	HC‐BD (*n* = 30)	BD (*n* = 32)	Group comparison test values
**Sociodemographic variables**			
Age	21.23 (1.68)	21.53 (1.76)	*t*(60) = 0.68, *p* = 0.50
Education level	13.27 (1.62)	13.60 (1.78)	*t*(53) = 0.73, *p* = 0.47
Gender, male (%)	50.00	53.00	*χ* ^2^ (1, 62) = 0.06, *p* = 0.81
**Alcohol‐related variables**			
Age at first alcohol consumption (years old)	15.28 (1.98)	14.44 (1.70)	*U* = 316.50, *p* = 0.03
Consumption per drinking occasion (in standard units)	1.98 (1.49)	9.22 (6.72)	*U* = 29.50, *p* < 0.001
AUDIT	2.10 (1.81)	13.84 (3.64)	*t*(46.06) = 16.25, *p* < 0.001
BD score	4.15 (3.89)	42.13 (16.38)	*U* = 0.00, *p* < 0.001
**Clinical variables**			
AIS	3.52 (2.68)	10.16 (23.63)	AIS; *U* = 306.00, *p* = 0.02
BDI	3.80 (2.94)	4.22 (2.67)	*U* = 428.50, *p* = 0.47
CTQ—Denial	1.07 (1.08)	0.81 (1.12)	*U* = 406.00, *p* = 0.27
CTQ—Emotional abuse	5.70 (1.53)	6.41 (2.38)	*U* = 385.50, *p* = 0.13
CTQ—Emotional neglect	8.37 (3.76)	9.34 (3.54)	*U* = 383.00, *p* = 0.17
CTQ—Physical abuse	5.03 (0.18)	5.09 (0.30)	*U* = 451.00, *p* = 0.35
CTQ—Physical neglect	5.37 (0.72)	5.78 (1.43)	*U* = 413.00, *p* = 0.26
CTQ—Sexual abuse	5.27 (1.48)	5.13 (0.42)	*U* = 453.50, *p* = 0.48
DES	14.76 (12.14)	16.33 (8.37)	*U* = 360.00, *p* = 0.09
DERS‐F—Total	74.86 (16.46)	79.69 (21.29)	*U* = 352.50, *p* = 0.11
DERS‐F—Awareness	16.31 (5.09)	16.34 (4.93)	*U* = 454.00, *p* = 0.89
DERS‐F—Clarity	10.41 (4.24)	9.13 (3.02)	*U* = 412.50, *p* = 0.46
DERS‐F—Goals	13.07 (4.62)	16.56 (5.51)	*t*(59) = 2.67, *p* = 0.01
DERS‐F—Impulse	8.52 (2.59)	10.63 (4.78)	*U* = 346.00, *p* = 0.09
DERS‐F—Nonacceptance	10.31 (5.29)	11.78 (4.49)	*U* = 318.50, *p* = 0.04
DERS‐F—Strategies	16.24 (4.70)	18.44 (6.00)	*t*(59) = 1.58, *p* = 0.12
PBCS	12.63 (2.68)	12.94 (2.42)	*U* = 474.00, *p* = 0.94
STAI‐A	31.07 (6.59)	33.59 (8.75)	*U* = 420.50, *p* = 0.41
STAI‐B	37.40 (9.09)	38.87 (9.23)	*t*(59) = 0.63, *p* = 0.53
TAS	47.67 (10.88)	46.88 (8.43)	*t*(60) = 0.32, *p* = 0.75
TAS—Difficulty describing feelings	13.90 (4.98)	13.41 (4.29)	*t*(60) = 0.42, *p* = 0.68
TAS—Difficulty identifying feelings	15.73 (4.66)	15.22 (3.70)	*t*(60) = 0.48, *p* = 0.63
TAS—Externally oriented thinking	18.03 (4.33)	18.25 (3.74)	*t*(60) = 0.21, *p* = 0.83

Abbreviations: AIS, Athens Insomnia Scale; AUDIT, Alcohol Use Disorders Identification Test; BDI, Beck Depression Inventory; CTQ, Childhood Trauma Questionnaire; DERS‐F, Difficulties in Emotion Regulation Scale‐French; DES, Dissociative Experiences Scale; PBCS, Private Body Consciousness subscale; STAI, State (‐A) and Trait (‐B) Anxiety Inventory; TAS, Toronto Alexithymia Scale.

Groups were matched on age, education level and gender ratio and differed solely on the alcohol‐related variable: BD had started drinking alcohol younger, consumed more alcohol per drinking occasion and had higher AUDIT and BD scores.

Concerning psychological and clinical variables assessment, BD reported more sleep problems, difficulties in engaging in goal‐related behaviours when experiencing negative emotions and nonacceptance of negative emotions.

The two groups did not differ significantly in terms of depressive symptoms, childhood trauma denial, emotional abuse, emotional neglect, physical abuse, physical neglect and sexual abuse. They did not significantly differ regarding dissociative symptoms experience, difficulties in regulating emotions, emotional awareness, emotional clarity, impulse control and access to emotion regulation strategies. HC‐BD and BD did not significantly differ regarding awareness and sensitivity to mostly uncomfortable physiological sensations and anxiety at the trait and state levels. Participants did not significantly differ regarding alexithymia levels, difficulty in describing feelings, identifying feelings and externally oriented thinking.

### 3.2 Interoceptive Accuracy

#### 3.2.1 SAUD and HC‐SAUD (Figure 1)

**FIGURE 1:**
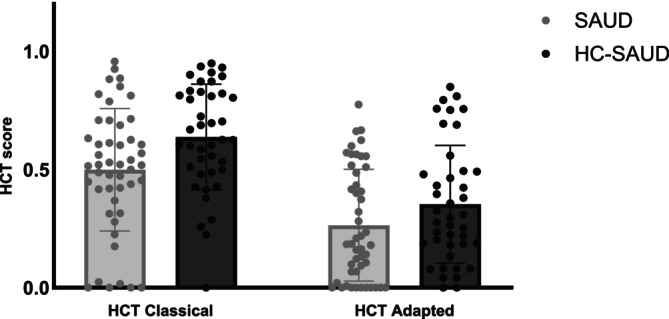
Heartbeat Counting Task (HCT) scores for patients with severe alcohol use disorder (SAUD) and paired healthy controls (HC‐SAUD), at the classical and adapted HCT.

Noteworthy, the SAUD/HC‐SAUD comparison identified a significant group effect for education level, with higher average education level for HC‐SAUD group (U = 334.50, *p* < 0.001), that correlated with interoceptive accuracy scores (HCT classical: r = 0.28, *p* = 0.008; HCT adapted: r = 0.20, *p* = 0.04), we thus added education level as a covariate in the mixed model.

The mixed model conducted on interoceptive accuracy scores revealed main effects of group (F(1, 87) = 6.17, *p* = 0.02) and task (F(1, 87) = 115.72, *p* < 0.001), but the group by task interaction effect was not significant (F(1, 87) = 1.06, *p* = 0.31). The main group effect showed lower scores for SAUD patients (M = 0.38; SE = 0.03) than HC‐SAUD (M = 0.49; SE = 0.03) and the main task effect revealed that participants had higher scores at the classical (M = 0.57; SE = 0.03) than adapted HCT (M = 0.31; SE = 0.03).

#### 3.2.2 BD and HC‐BD (Figure 2)

**FIGURE 2:**
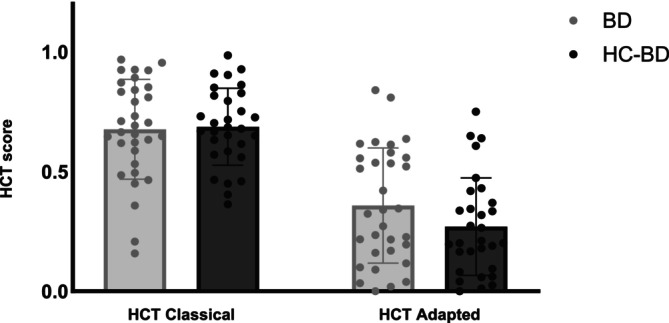
Heartbeat Counting Task (HCT) scores for binge drinkers (BD) and paired healthy controls (HC‐BD), at the classical and adapted HCT.

The mixed model conducted on interoceptive accuracy scores found only the main task effect to be significant (F(1, 60) = 164.27, *p* < 0.001), while neither the main group (F(1, 60) = 0.79, *p* = 0.38) nor the group by task interaction effects were significant (F(1, 60) = 2.97, *p* = 0.09). Similarly to the HC‐SAUD and SAUD results, participants had higher scores at the classical (M = 0.68; SE = 0.03) than adapted HCT (M = 0.31; SE = 0.03).

### 3.3 Self‐Reports

For clarity's sake, hereinafter are reported solely significant correlations, the correlation matrix can be found in the Tables S1 and S2.

#### 3.3.1 SAUD

We found a significant correlation between interoceptive accuracy scores at the classical and adapted HCT (r = 0.56, *p* < 0.001).

#### 3.3.2 HC‐SAUD

We found one significant correlation, between the performance at the classical and adapted HCT (r = 0.56, *p* < 0.001).

#### 3.3.3 BD/HC‐BD

We found no significant correlation with neither the classical nor adapted HCT scores that survived Benjamini–Hochberg correction, including between the performance at the classical and adapted HCT (r_BD_ = 0.39, *p*_*BD*_ = 0.03; r_HCBD_ = 0.42, *p*_*HCBD*_ = 0.02).

### 3.4 Time Estimation

#### 3.4.1 SAUD

We found significant correlations between the number of seconds reported per minute and the number of heartbeats reported per minute at the classical HCT (r = 0.31, *p* = 0.03) but not with the number of heartbeats reported per minute at the adapted HCT (r = 0.23, *p* = 0.12).

#### 3.4.2 HC‐SAUD

We also found significant correlations between the number of seconds reported per minute and the number of heartbeats reported per minute at the classical HCT (r = 0.35, *p* = 0.02), but not with the number of heartbeats reported at the adapted HCT (r = 0.17, *p* = 0.28).

#### 3.4.3 BD

We found no correlation between the number of seconds reported per minute and the number of heartbeats reported per minute at the classical (r = 0.28, *p* = 0.13) or adapted (r = 0.24, *p* = 0.18) HCT.

#### 3.4.4 HC‐BD

We found no correlation between seconds reported per minute and heartbeats reported per minute at the classical (r = 0.09, *p* = 0.65) or adapted (r = 0.32, *p* = 0.08) HCT.

## 4 Discussion

Theoretical models and preclinical research posit that interoception plays a role in alcohol use disorder onset and maintenance. However, to date, the few reliable behavioural evidence in SAUD population is mixed and relies mainly on the HCT, a task severely criticized in the interoception field [19]. We explored the validity of this measure of interoceptive accuracy (HCT) across two critical stages of the problematic alcohol use continuum, namely, in clinical (SAUD) and subclinical (BD) alcohol use disorder.

First, we compared performance at the classical and adapted versions of the HCT. We found, in both clinical and subclinical samples, higher HCT scores at the classical than adapted task. This large difference further supports prior concerns about the lack of construct validity of the classical HCT, as it, again, highlights the large role of non‐interoceptive contributions to task performance. We also found that HC‐SAUD had overall higher scores than SAUD on both HCT versions—an effect that was not found in the subclinical counterpart. One possible explanation for this group difference is that HC participants may be better at detecting their heartbeats (i.e., may present higher interoceptive accuracy) than SAUD patients—an explanation that concurs with the nonsignificant group by task interaction.

Such interpretation suggests that non‐interoceptive strategies, including guessing, were effectively removed in the adapted version of the task. This is supported by the lack of significance between reported heartbeats and reported seconds in this version (vs. the classical) of the task. Recent research, however, indicates that a significant proportion of participants still estimate their heart rate even when explicitly instructed not to [47], which calls this assumption into question. Indeed, if a proportion of participants in each group continues to guess their heart rate to perform the task, group differences could still be explained by guessing abilities rather than interoceptive accuracy abilities. Furthermore, besides guessing and detection ability, other contributing factors have been identified in the HCT: decision threshold (i.e., the amount of evidence needed for each individual to report that a heartbeat was felt) [19] and cardiac signal intensity [14]. These contributing factors could also account for group differences. Two main conclusions can thus be drawn from this first objective: (1) the classical HCT is strongly influenced by non‐interoceptive strategies in both patients with SAUD, BD and HCs, as illustrated by strong performance drop when using the adapted HCT (i.e., when explicitly requesting participants to avoid guessing or relying on non‐interoceptive cues); (2) SAUD patients report less heartbeats than HC–a group effect that could be underlid by multiple interoceptive and non‐interoceptive‐related factors which, nonetheless, cannot be explored with the HCT.

Second, we examined how HCT performance relates to self‐reported psychological and clinical variables theoretically associated with SAUD and interoceptive accuracy [23, 26, 29, 30, 31]. We found no significant correlations with neither task version. Although our sample size calls for caution, this finding is consistent with recent meta‐analyses identifying no relation between performance at the HCT and mental health‐related variables [21, 22]. Such null results further question the construct validity of both HCT versions. Moreover, this result further underlines the need to cease considering the HCT as a ‘gold standard’ in the assessment of interoception in SUD, as its use leads to problematic insights. Would the HCT be valid, our results would suggest no association between cardiac accuracy of SAUD patients and BD participants and, for example, the self‐reported ability to regulate emotions (DERS [40]). This is hard to reconcile with the fact that there are indeed reports of an association between interoception, emotion regulation and mental health outcomes such as SUD [48]. Moreover, there are strong theoretical expectation that cardiac (and, more generally, interoceptive) accuracy helps with emotion regulation [49]. More broadly, we would like to question the sole reliance on one bodily system (i.e., cardiac) to probe the role of interoceptive accuracy in SUD. This is problematic especially when considering that measures targeting different sensory axes have low convergence [16]. Thus, in addition to the need to stop using the HCT in SUD, there is a need to develop valid tools to measure interoception across bodily systems in SUD. This will allow to identify the most relevant bodily system(s) in SUD. Furthermore, current interoceptive models include many other features beyond accuracy [50]. These other features (e.g., interoceptive attention or interpretation) may be at least as relevant for SUD. Future research should thus explore other features of interoception in SUD.

Finally, we examined the contribution of time estimation in HCT performance [20]. The association between reported numbers of heartbeats at the classical HCT and seconds counted during the time estimation task further supports that a significant proportion of participants rely on time‐based strategies. Conversely, such correlation was not found for BD and HC‐BD. We did find, however, a significant decrease between accuracy scores at the classical and adapted HCT for the subclinical sample. One explanation for this lack of correlation could be that, while BD and HC‐BD did rely on non‐interoceptive strategies, time estimation was not the one used. It could be that participants relied on their own knowledge about heart rate [20]. In sum, although the effect of instruction modification suggests that all samples estimated their heart rate in the classical HCT, the correlations with time estimation indicate that BD and HC‐BD were not using the same estimation strategies.

Our study, while providing further evidence for the lack of validity of the HCT, is not without limitations. First, we focused on one non‐interoceptive strategy, without asking participants about the strategies they used [28]. For instance, while we infer that no estimation strategy is used in the adapted HCT, it would have been useful to directly ask participants to back up this claim as we did not explore all the variables that underpinned HCT. Moreover, we cannot exclude that our study results are task‐dependent and, hence, that other measures exploring the validity of the HCT would have led to different results than the one found here. Second, this study had a single‐blind design. This could have led the experimenter to unknowingly exert an influence on participants [51]. Since a decrease in performance between tasks was expected [28], the experimenter may have involuntarily induced participants to perform accordingly. Finally, while the adapted HCT was used as a mean to explore the validity of Schandry's task [18], we offered no valid alternative to interoceptive accuracy assessment, which prevented us to test the convergent validity of the HCT. However, this remains impossible due to the absence of gold standard task validly assessing cardiac interoception.

To conclude, we explored the validity of two versions of the HCT in both BD and SAUD. We found that the classical HCT has major validity issues, in both clinical and subclinical SUD as it (1) strongly overestimates interoceptive accuracy (as compared to the adapted HCT); (2) is not related to psychological and clinical variables theoretically related to interoceptive abilities; (3) is significantly associated with time estimation. On the one hand, SAUD patients and HC‐SAUD differed on HCT performance no matter the instructions used. Results suggest also that both groups relied on time estimation in the classical but not the adapted HCT. On the other hand, BD do not appear to use such time estimation strategies (but might use other non‐interoceptive cues, as shown by the performance drop between classical and adapted HCT). This intergroup variability in the respondents' reliance of non‐interoceptive strategies complicates the interpretation of results found in previous studies using the HCT in SAUD. We hence raise awareness on validity issues plaguing the HCT in SUD research, in the few studies exploring conscious interoception in this field. It is important to note that, while the adapted version of the HCT reduces the bias related to non‐interoceptive strategies, it does not constitute a reliable alternative to measure interoceptive accuracy. Indeed, this adapted version was not developed as a mean to propose a valid interoceptive measure (but rather as a tool to test the influence of non‐interoceptive variables on original HCT performance [28]) and still presents strong limits, ending up in a low validity [43]. Our results thus show that the HCT, in its original or adapted version, does not offer researchers and clinicians a reliable and valid way to measure interoception accuracy. They hence call for the development of validated interoceptive measures to more reliably advance the study of interoceptive processes at play in SUD. We recommend that future research adapt their interpretations of the HCT scores based on the potential contaminators of the task [52] or administer alternative tasks that are shown to be more valid [14]. However, we would like to reemphasize that to date, no gold standard has yet emerged. Researchers are thus encouraged to keep themselves updated about upcoming studies demonstrating the validity of new tasks. Alternatively, we also encourage researchers to investigate other features of interoception (e.g., self‐report dimensions), which may be more validly assessed.

## Conflicts of Interest

PM (Senior Research Associate), PB (PDR Research Associate), OC (Full Professor), OL (Research Director) and MLF (Postdoctoral Research Associate) are funded by the Belgian Fund for Scientific Research (FRS‐FNRS, Belgium). MLF is also funded by the MSCA Individual Postdoctoral Fellowship. OD (Postdoctoral Research Associate) is funded by the Swiss National Science Foundation (SNSF) and the Swiss Foundation for Alcohol Research (SSA).

## Supporting information


**Table S1.** Correlation matrix between interoceptive accuracy scores at the classical heartbeat counting task (HCT) and self‐reported psychopathological variables.
**Table S2.** Correlation matrix between interoceptive accuracy scores at the adapted heartbeat counting task (HCT) and self‐reported psychopathological variables.

## Data Availability

All data are available from the Open Science Framework (OSF): https://osf.io/ysd2e/?view_only=65a69691a2cb40708fa2af51aef40d87.
