# Supplementary material for: Validity Concerns About the Heartbeat Counting Task Extend to Alcohol Use disorder: Evidence From Subclinical and Clinical Samples
Source: Addict Biol. 2025 May 1;30(5):e70032. doi: 10.1111/adb.70032 (PMC12044521; doi:10.1111/adb.70032)
Supplement: Supplementary file 1 — Table S1. Correlation matrix between interoceptive accuracy scores at the classical heartbeat counting task (HCT) and self‐reported psychopathological variables. Table S2. Correlation matrix between interoceptive accuracy scores at the adapted heartbeat counting task (HCT) and self‐reported psychopathological variables. [file ADB-30-e70032-s001.docx]

**Supplementary Table 1.** Correlation matrix between interoceptive accuracy scores at the classical heartbeat counting task (HCT) and self-reported psychopathological variables.

|  | Interoceptive accuracy at the classical HCT | | | | | | | |
| --- | --- | --- | --- | --- | --- | --- | --- | --- |
|  | HC-SAUD (n=41) | | SAUD (n=48) | | HC-BD (n=30) | | BD (n=32) | |
|  | r | *p-value* | r | *p-value* | r | *p-value* | r | *p-value* |
| Interoceptive accuracy at the adapted HCT | **0.56** | **<0.001** | **0.56** | **<0.001** | 0.42 | 0.02 | 0.39 | 0.03 |
| AIS | -0.17 | 0.33 | -0.02 | 0.91 | 0.11 | 0.56 | 0.00 | 0.99 |
| BDI | -0.02 | 0.88 | -0.06 | 0.69 | -0.03 | 0.86 | 0.08 | 0.67 |
| CTQ – Denial | 0.14 | 0.40 | -0.03 | 0.83 | 0.06 | 0.76 | -0.05 | 0.77 |
| CTQ – Emotional abuse | 0.08 | 0.63 | -0.04 | 0.78 | 0.03 | 0.88 | 0.09 | 0.61 |
| CTQ – Emotional neglect | 0.07 | 0.68 | -0.02 | 0.89 | -0.13 | 0.50 | 0.09 | 0.63 |
| CTQ – Physical abuse | -0.06 | 0.74 | -0.13 | 0.39 | -0.23 | 0.23 | 0.02 | 0.93 |
| CTQ – Physical neglect | -0.01 | 0.96 | -0.10 | 0.51 | 0.03 | 0.87 | 0.00 | 0.98 |
| CTQ – Sexual abuse | 0.03 | 0.87 | 0.13 | 0.38 | 0.17 | 0.38 | -0.14 | 0.45 |
| DES | -0.00 | 0.98 | -0.18 | 0.23 | 0.01 | 0.97 | 0.06 | 0.75 |
| DERS-F – Total | -0.18 | 0.27 | -0.09 | 0.56 | 0.00 | 0.99 | 0.11 | 0.54 |
| DERS-F – Awareness | -0.16 | 0.33 | -0.31 | 0.03 | -0.26 | 0.18 | -0.11 | 0.55 |
| DERS-F – Clarity | -0.19 | 0.24 | -0.25 | 0.09 | -0.15 | 0.43 | 0.15 | 0.42 |
| DERS-F – Goals | -0.14 | 0.38 | 0.12 | 0.43 | 0.07 | 0.72 | 0.12 | 0.50 |
| DERS-F – Impulse | -0.20 | 0.23 | -0.13 | 0.38 | 0.00 | 1.00 | -0.12 | 0.52 |
| DERS-F – Nonacceptance | -0.16 | 0.31 | 0.11 | 0.48 | -0.12 | 0.54 | 0.12 | 0.51 |
| DERS-F – Strategies | -0.01 | 0.93 | 0.05 | 0.76 | 0.08 | 0.69 | 0.19 | 0.29 |
| PBCS | -0.25 | 0.14 | 0.38 | 0.01 | -0.12 | 0.51 | 0.02 | 0.89 |
| STAI-A | -0.39 | 0.01 | -0.04 | 0.81 | -0.25 | 0.18 | 0.05 | 0.77 |
| STAI-B | -0.21 | 0.19 | 0.05 | 0.74 | -0.14 | 0.47 | 0.08 | 0.69 |
| TAS | -0.13 | 0.44 | -0.14 | 0.35 | -0.08 | 0.69 | 0.27 | 0.13 |
| TAS – Difficulty describing feelings | -0.11 | 0.51 | -0.16 | 0.27 | -0.03 | 0.87 | 0.32 | 0.08 |
| TAS – Difficulty identifying feelings | -0.10 | 0.55 | -0.20 | 0.17 | -0.05 | 0.78 | 0.19 | 0.31 |
| TAS – Externally-oriented thinking | -0.13 | 0.44 | 0.02 | 0.91 | -0.24 | 0.20 | 0.15 | 0.40 |

*Note.* AIS: Athens Insomnia Scale; AUDIT: Alcohol Use Disorders Identification Test; BD: Binge Drinkers; BDI: Beck Depression Inventory; CTQ: Childhood Trauma Questionnaire; DES: Dissociative Experiences Scale; DERS-F: Difficulties in Emotion Regulation Scale-French; DSM: Diagnostic and Statistical Manual of Mental Disorders; HC-BD: Healthy Controls paired with BD; HC-SAUD: Healthy Controls paired with SAUD patients; PBCS: Private Body Consciousness subscale; SAUD: Severe Alcohol Use Disorder; STAI: State and Trait Anxiety Inventory; TAS: Toronto Alexithymia Scale.

**Supplementary Table 2.** Correlation matrix between interoceptive accuracy scores at the adapted heartbeat counting task (HCT) and self-reported psychopathological variables.

|  | Interoceptive accuracy at the adapted HCT | | | | | | | |
| --- | --- | --- | --- | --- | --- | --- | --- | --- |
|  | HC-SAUD (n=41) | | SAUD (n=48) | | HC-BD (n=30) | | BD (n=32) | |
|  | r | *p-value* | r | *p-value* | r | *p-value* | r | *p-value* |
| AIS | -0.14 | 0.40 | 0.07 | 0.62 | 0.28 | 0.14 | 0.15 | 0.41 |
| BDI | -0.18 | 0.25 | -0.02 | 0.90 | -0.00 | 0.99 | -0.11 | 0.54 |
| CTQ – Denial | -0.02 | 0.91 | 0.04 | 0.79 | -0.12 | 0.52 | 0.06 | 0.75 |
| CTQ – Emotional abuse | 0.12 | 0.50 | 0.09 | 0.56 | 0.12 | 0.53 | 0.14 | 0.46 |
| CTQ – Emotional neglect | 0.14 | 0.43 | -0.03 | 0.86 | 0.06 | 0.74 | 0.22 | 0.23 |
| CTQ – Physical abuse | -0.14 | 0.40 | 0.07 | 0.67 | 0.03 | 0.87 | 0.09 | 0.64 |
| CTQ – Physical neglect | 0.04 | 0.81 | -0.14 | 0.34 | 0.10 | 0.61 | 0.26 | 0.15 |
| CTQ – Sexual abuse | -0.01 | 0.97 | 0.06 | 0.68 | -0.09 | 0.65 | -0.00 | 0.98 |
| DES | 0.21 | 0.22 | 0.05 | 0.77 | -0.01 | 0.97 | -0.14 | 0.45 |
| DERS-F – Total | -0.18 | 0.27 | 0.02 | 0.88 | -0.16 | 0.40 | -0.33 | 0.06 |
| DERS-F – Awareness | -0.28 | 0.08 | -0.06 | 0.70 | 0.13 | 0.50 | 0.20 | 0.28 |
| DERS-F – Clarity | -0.15 | 0.37 | -0.02 | 0.87 | 0.19 | 0.31 | -0.06 | 0.75 |
| DERS-F – Goals | -0.06 | 0.72 | 0.18 | 0.22 | -0.31 | 0.11 | -0.29 | 0.11 |
| DERS-F – Impulse | -0.06 | 0.69 | -0.00 | 1.00 | -0.39 | 0.04 | -0.24 | 0.18 |
| DERS-F – Nonacceptance | -0.11 | 0.51 | 0.04 | 0.79 | -0.25 | 0.19 | -0.12 | 0.51 |
| DERS-F – Strategies | 0.04 | 0.82 | 0.01 | 0.95 | -0.18 | 0.35 | -0.25 | 0.17 |
| PBCS | -0.09 | 0.60 | 0.38 | 0.01 | -0.17 | 0.37 | 0.05 | 0.80 |
| STAI-A | -0.32 | 0.04 | -0.08 | 0.61 | -0.19 | 0.31 | -0.03 | 0.87 |
| STAI-B | -0.15 | 0.36 | 0.06 | 0.70 | -0.14 | 0.47 | -0.16 | 0.40 |
| TAS | -0.20 | 0.24 | 0.00 | 0.99 | 0.23 | 0.23 | -0.02 | 0.89 |
| TAS – Difficulty describing feelings | -0.20 | 0.25 | 0.13 | 0.39 | 0.23 | 0.22 | 0.07 | 0.71 |
| TAS – Difficulty identifying feelings | -0.08 | 0.65 | -0.05 | 0.75 | 0.07 | 0.72 | -0.09 | 0.61 |
| TAS – Externally-oriented thinking | -0.25 | 0.14 | 0.08 | 0.61 | 0.09 | 0.62 | 0.19 | 0.29 |

*Note.* AIS: Athens Insomnia Scale; AUDIT: Alcohol Use Disorders Identification Test; BD: Binge Drinkers; BDI: Beck Depression Inventory; CTQ: Childhood Trauma Questionnaire; DES: Dissociative Experiences Scale; DERS-F: Difficulties in Emotion Regulation Scale-French; DSM: Diagnostic and Statistical Manual of Mental Disorders; HC-BD: Healthy Controls paired with BD; HC-SAUD: Healthy Controls paired with SAUD patients; PBCS: Private Body Consciousness subscale; SAUD: Severe Alcohol Use Disorder; STAI: State and Trait Anxiety Inventory; TAS: Toronto Alexithymia Scale.
